# The impact of a brief lifestyle intervention delivered by generalist community nurses (CN SNAP trial)

**DOI:** 10.1186/1471-2458-13-375

**Published:** 2013-04-22

**Authors:** Mark F Harris, Bibiana C Chan, Rachel A Laws, Anna M Williams, Gawaine Powell Davies, Upali W Jayasinghe, Mahnaz Fanaian, Neil Orr, Andrew Milat

**Affiliations:** 1 Centre for Primary Health Care and Equity, School of Public Health and Community Medicine, University of New South Wales, Sydney, NSW, 2052, Australia

**Keywords:** Primary health care, Lifestyle behaviours, Smoking, Nutrition, Alcohol, Physical activity, Community nursing

## Abstract

**Background:**

The risk factors for chronic disease, smoking, poor nutrition, hazardous alcohol consumption, physical inactivity and weight (SNAPW) are common in primary health care (PHC) affording opportunity for preventive interventions. Community nurses are an important component of PHC in Australia. However there has been little research evaluating the effectiveness of lifestyle interventions in routine community nursing practice. This study aimed to address this gap in our knowledge.

**Methods:**

The study was a quasi-experimental trial involving four generalist community nursing (CN) services in New South Wales, Australia. Two services were randomly allocated to an ‘early intervention’ and two to a ‘late intervention’ group. Nurses in the early intervention group received training and support in identifying risk factors and offering brief lifestyle intervention for clients. Those in the late intervention group provided usual care for the first 6 months and then received training. Clients aged 30–80 years who were referred to the services between September 2009 and September 2010 were recruited prior to being seen by the nurse and baseline self-reported data collected. Data on their SNAPW risk factors, readiness to change these behaviours and advice and referral received about their risk factors in the previous 3 months were collected at baseline, 3 and 6 months. Analysis compared changes using univariate and multilevel regression techniques.

**Results:**

804 participants were recruited from 2361 (34.1%) eligible clients. The proportion of clients who recalled receiving dietary or physical activity advice increased between baseline and 3 months in the early intervention group (from 12.9 to 23.3% and 12.3 to 19.1% respectively) as did the proportion who recalled being referred for dietary or physical activity interventions (from 9.5 to 15.6% and 5.8 to 21.0% respectively). There was no change in the late intervention group. There a shift towards greater readiness to change in those who were physically inactive in the early but not the comparison group. Clients in both groups reported being more physically active and eating more fruit and vegetables but there were no significant differences between groups at 6 months.

**Conclusion:**

The study demonstrated that although the intervention was associated with increases in advice and referral for diet or physical activity and readiness for change in physical activity, this did not translate into significant changes in lifestyle behaviours or weight. This suggests a need to facilitate referral to more intensive long-term interventions for clients with risk factors identified by primary health care nurses.

**Trial registration:**

ACTRN12609001081202

## Background

In Australia, chronic diseases such as heart disease and diabetes are the leading causes of death and disability [1]. The risk factors for these conditions include risk behaviours (in smoking, nutrition, alcohol and physical activity) and overweight (SNAPW). These are prevalent in the community, with over 90% of adults not consuming the recommended five serves of vegetables per day, over half not consuming adequate amounts of fruit, 62% overweight or obese, one third, physically inactive, one in five smoke and 21% drink alcohol at levels which pose a risk to their health [2].

Primary health care (PHC) is an important setting for addressing lifestyle risk factors because of its accessibility, continuity, and comprehensiveness of the care provided [3]. There is evidence that clients expect to receive lifestyle intervention from PHC clinicians [4]. Lifestyle interventions delivered in PHC are effective in helping clients to stop smoking [5], reduce ‘at-risk alcohol’ consumption [6], improve weight, diet and physical activity levels [7-12]. The 5As (assess, advise (including motivational interviewing) and agree on goals, assist (including referral), and arrange (follow up) have been developed as a framework for addressing these risk factors in clinical practice [13,14].

In NSW, generalist community nurses frequently see clients in their own home, providing care for patients recently discharged from hospital, the aged and those with chronic diseases. Although the traditional community nursing model of practice includes health promotion activities, community nursing services have increasingly tended to provide shorter term more clinically focused services to individual clients [15,16]. Our previous research has shown that community health nurses consider the provision of lifestyle intervention appropriate to their role and it is well accepted by clients [17]. However, few studies have evaluated the effectiveness of lifestyle interventions provided by community nurses in routine practice [18-21]. The aim of this study was to evaluate the impact of a brief lifestyle intervention delivered by community health nurses as part of their routine practice on changes in clients’ SNAPW risk factors.

## Methods

### Study design and setting

This study was conducted in four general community nursing services in New South Wales, Australia. Services were recruited via an expression of interest mailed to all Area Health Services (AHS) in NSW (n = 8). The design was quasi-experimental, with the services randomly allocated to an ‘early intervention’ (EI) group or ‘late intervention’ (LI) (comparison) group. EI services were provided with training and support for nurses in identifying clients with high risk and offering brief SNAPW intervention during routine consultations. The protocol for the study has been previously described [22].

### Intervention

The intervention was designed and implemented on two levels: (a) service level and (b) client level.

#### Service-level intervention

The service-level intervention was delivered by University staff and consisted of four components:

• A 1-day training program in the assessment and management of the SNAPW risk factors (including motivational interviewing) for participating community nurses delivered by the research team in conjunction with local providers. The training included the use of role-plays with simulated clients (actors), group discussions and activities;

• Integration of standardised screening tools and prompts for SNAPW risk factors into the service-specific assessment processes used by the nurses in the management of clients;

• Development and distribution of a local service referral directory to each community nursing team to promote referral of clients for ongoing specialist management or more / ongoing intensive lifestyle intervention; and

• Provision of client resources to all participating nurses. The resources included a written guide for nurses, written action plans for use with clients on each SNAPW risk factor, tape measures for measuring waist circumference and pedometers for loan to clients to encourage self-monitoring of physical activity**.**

A nurse from each of the EI sites was seconded to work with the research team to develop the intervention and to support its implementation at the local level.

#### Client-level intervention

The client-level intervention was provided by the participating nurses. The goals of the clinical intervention were to achieve and maintain lifestyle changes consistent with current Australian recommendations [23]:

• Moderate physical activity for at least 30 minutes/day, including walking, jogging, swimming, aerobic activity, ball games, skiing, with circuit-type resistance training if possible, twice a week;

• A diet low in saturated fats, sucrose and salt with increased portions of vegetables and fruit per day (up to seven portions) in order to achieve a diet where the percentage of energy from carbohydrates = 50%, saturated fats <10% (and total fats < 30%, protein 1 g/kg ideal body weight per day, fibre 15 g/1000 kcal);

• Weight reduction (if overweight) of ≥ 5 kg or 5% of body weight;

• Smoking cessation (if smoker);

• Limit alcohol intake (if drinking) to ≤ 2 drinks / day, including 1–2 alcohol-free days/week.

**Figure 1:**
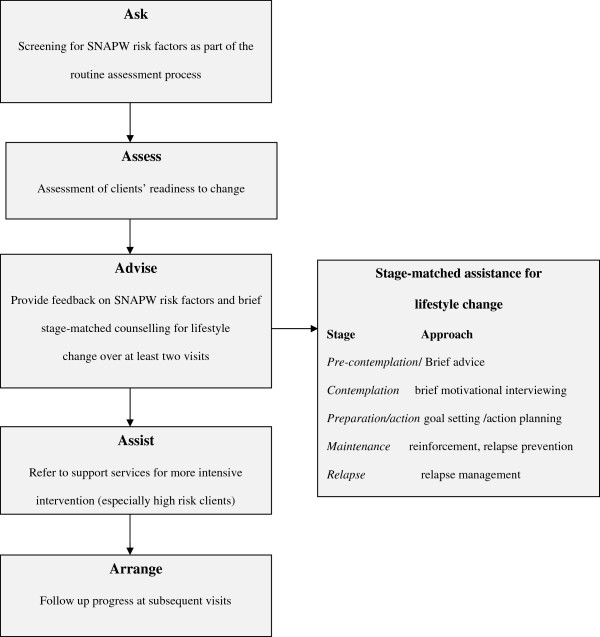
5As model of brief lifestyle intervention.

The nurses assessed clients’ lifestyle risk factors and then provided brief educational intervention tailored to their readiness to change, based on the 5As Model [3] (Figure 1), for one or more SNAPW risk factors. This occurred over two or more visits. Clients who were ‘at risk’ (those who were obese, smoked or who had multiple risk behaviours or illnesses arising from them) and who were in the contemplation or action stages of change were referred to specialist providers for more intensive intervention.

### Late intervention

Two of the four CN services were randomly allocated to the LI group. Late intervention services provided usual care for 18 months. After all data ad been collected the service level intervention was introduced into these services as well.

### Client recruitment

**Figure 2:**
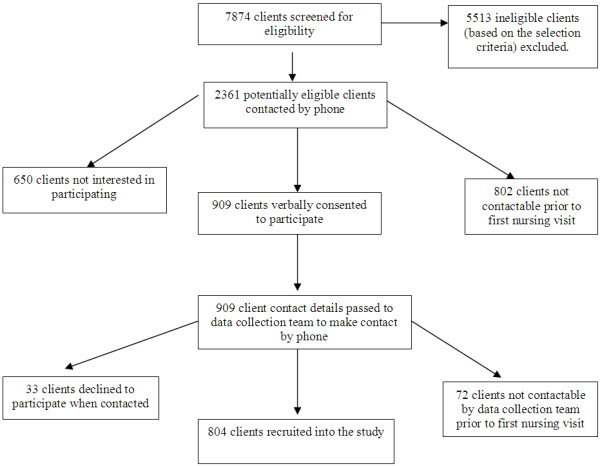
Client recruitment process and baseline data collection.

Between September 2009 and September 2010 clients who met the selection criteria and who had been referred to participating services (Table 1) were invited to participate in the study. Potential participants were contacted by phone on the day of referral (where possible) by trained local recruitment officers. The client recruitment process is outlined in Figure 2.

**Table 1:** Selection criteria for recruiting clients to participate in the trial

**Types**	**Selection criteria**
**Inclusion criteria**	* Clients referred to community nursing services
	* Age 30–80 years
	* Able to read and understand English at a level that enables the client to participate in a telephone-administered survey and to understand the participant information sheet.
**Exclusion criteria**	* Palliative care clients.
	* Clients receiving only one- visit or occasion of service.
	* Clients with significant cognitive impairment (unable to complete telephone-administered survey).
	* Clients currently receiving help in changing their lifestyle from a health professional (other than their GP) such as a dietitian or exercise physiologist.
	* Clients currently attending a chronic disease management program such as cardiac rehabilitation, diabetes education program.
	* Clients who have attended the generalist community nursing service in the previous 6 months (and therefore may have already received lifestyle intervention).

### Study outcomes, measurements and data collection

The study outcomes, measurement tools and timeframe for data collection are summarised in Table 2. The measurement tools were validated in other research [24-26]. The diet, physical activity, alcohol and weight outcomes were continuous measures. Diet score was the total number of serves of fruit and vegetables per day up to a maximum of 7. The physical activity score combined assessment of duration of vigorous and moderate physical activity (scored 0–8, <4 considered at risk) [27]. Alcohol was the average number of standard drinks per day. Smoking status was a categorical variable (smoker or non-smoker). Mediator variables included change in clients’ ‘readiness to change’ lifestyle behaviours [28]. Process measures included change in clients’ recall of advice or referral over the previous 3 months. Data were collected from a 15-minute telephone-administered survey conducted with clients at baseline (prior to first nurse visit) and at 3 and 6 months. The telephone survey was conducted by trained independent data collectors blinded to the group allocation (EI or LI) groups.

**Table 2:** Study outcomes and measurement

**Outcomes**	**Measurement**
Change in mean physical activity score	Brief validated physical activity tool [24]
Change in mean alcohol intake score	Validated AUDIT-C tool [25]
Change in mean number of serves of fruit and vegetables	Validated questions from the NSW Health survey [26]
Mean weight change	Self report
Change in smoking status	Self report
Change in adequate levels of physical activity	Self report
Change in ‘at risk’ alcohol consumption	Self report
Change consumption of > =2 serves of fruit per day	Self report
Change in consumption of > =5 serves of vegetables per day	Self report
Progression in stages of change	On five point intentions scales [28]
At risk clients offered evidence-based advice to modify their risk factors	Recall over previous 3 months
At risk clients offered evidence-based referral to modify their risk factors	Recall over previous 3 months

Collected by client telephone survey at baseline, 3 and 6 months.

### Statistical analysis

#### Power and sample size calculation

The a priori sample size was 400 clients per group (n = 800). This was calculated based on estimates of change in mean risk scores of self-reported measures of lifestyle risk factors. This was sufficient based on a standard deviation from previous research [29], design effect of 1.8 and loss to follow up of 20% to detect the following changes in mean risk scores:-

• 1 portion of fruit and vegetables per day (based on sd 2.02)

• 1 unit of physical activity score (based on sd 2.13)

• 5 kg of self-reported weight loss (based on sd 14.95)

#### Analysis

Univariate comparisons were made within group between baseline and 3 months and between groups for receipt of advice and referral. Change in readiness to change was categorised at 6 months and compared between groups. Statistical tests included t test for continuous variables and chi square test for categorical variables.

Change in clients’ lifestyle risk factors between the EI and LI (comparison) groups were evaluated using multilevel models which included a number of patient level covariates thought to possibly influence the outcomes [30]. Three repeated measures of SNAPW were compared within clients [31]. Multilevel linear regression analysis was conducted on physical activity score, diet score and weight. In the first model three levels were fitted which included: service (level 3), client (level 2) and time (level 1). The variance between services was found not to be significant. For each risk factor at 6 months, a two level regression model was fitted. This included the time and client as levels adjusting for baseline risk, intervention, linear time (0 = baseline; 1 = 3 months; 2 = 6 months), gender, age, employment status, reason for referral, mental health and physical health status, number of risk factors and physical limitation. The multilevel statistical models were fitted using MLwiN version 2.25 [32].

### Ethics

The project was approved by the Hunter New England Human Research Ethics Committee (Ref No 08/10/15/4.03), and ratified by the University of New South Wales Human Research Ethics Committee (HREC) and the Human Research Ethics Committees in each of the participating Area Health Services. The study was conducted in compliance with this Committees regulations and the Helsinki declaration. All participants provided full informed written consent for publication of findings from this research.

## Results

### Baseline characteristics

A total of 804 clients were recruited from 2361 potentially eligible clients (34.1%), 425 in the EI group and 379 in the LI group (Figure 2). Just under half (49.3%) were female, 67.1% were 60 years of age or over and 53.1% were retired from paid work. Few participants spoke a language other than English or were of Aboriginal or Torres Strait Islander descent (Table 3). There were no significant differences in age and gender between those who accepted and those who declined to participate or between those in the EI and LI groups (Table 3).

**Table 3:** Characteristics of CN SNAPW trial clients at baseline

**Characteristics**	**Total (n = 804)**		**Early interv (n = 425)**		**Late interv (379)**	
	**N**	**%**	**N**	**%**	**N**	**%**
Female	396	49.3	214	50.4	182	48.0
Aboriginal/ Torres Strait Islander	4	0.5	2	0.5	2	0.5
Language other than English	35	4.4	18	4.2	17	4.5
Employed	215	26.7	115	27.1	100	26.4
Unable to work (long-term sickness/ disability)	109	13.6	50	11.8	59	15.6
Retired from paid work	419	53.1	229	53.9	190	50.1
**Age (yrs)**						
30-39 yrs	44	5.5	22	5.2	22	5.8
40-49	78	9.7	44	10.4	34	9.0
50-59	142	17.7	76	18.0	66	17.4
60-69	256	31.9	136	32.2	120	31.7
≥ 70	280	35.2	143	34.3	137	36.1
***Self-rated health status*** Poor or Fair	308	38.3	158	37.2	150	39.6
***Self-rated mental health status: Downhearted or blue***						
Most to all of the time	102	12.7	49	11.5	53	14.0
**Health conditions**						
Hypertension	395	49.1	225	52.9	170	44.9
Arthritis	277	34.5	155	36.5	122	32.2
High cholesterol	239	29.7	132	31.1	107	28.2
Cancer	213	26.5	123	28.9	90	23.7
Diabetes	185	23.0	102	24.0	83	21.9
Depression	132	16.4	66	15.5	66	17.4
Heart disease	132	16.4	55	15.9	55	17.1
no risk factors	18 (2.2%)		11 (2.6%)		7 (1.8%)	
1 risk only	147 (18.3%)		76 (17.9%		71 (18.7%)	
2 risks	328 (40.2%)		164 (38.6%)		159 (42.0%)	
3 risks	215 (26.7%)		120 (28.2		95 (25.1%)	
4 risks	92 (11.4%)		50 (11.8%)		42 (11.1%)	
5 risks	9 (1.1%)		4 (0.9)		5 (1.3)	
< 2 serves of fruit (n = 801)	336 (41.9%)		174 (40.9%)		162 (42.7%)	
<5 serves of veg (n = 796)	672 (84.4%)		352 (82.8%)		320 (84.4%)	
At risk alcohol consumption ^a^ (n = 804)	297 (36.9%)		159 (37.4%)		138 (36.4%)	
Smokers (n = 802)	138 (17.2%)		74 (17.5%)		65 (17.2%)	
Overweight (OW) (n = 785)	263 (33.5%)		123 (29.8%)		140 (37.6%)	
Obese (n = 785)	318 (40.5%)		182 (44.1%)		136 (36.6%)	
OW or obese (n = 785)	581 (74.0%)		305 (71.8%)		276 (72.8%)	
Unable to do physical activity (PA)^b^ (n = 793)	375 (47.3%)		196 (46.2%)		179 (47.2%)	
Able to do PA but inadequate (n = 418)	211 (50.5%)		120 (31.2%)		91 (25.9%)	

^a^ The 2009 national alcohol guidelines for alcohol consumption are available at: http://www.health.gov.au/internet/alcohol/publishing.nsf/Content/guide-adult.

^b^ Those with major physical limitations which (a) limited their ability to engage in physical activity a lot and (b) were estimated to last for more than 4 weeks, or unsure.

The majority (61.6%) of clients rated their own health as ‘*good, very good or excellent’* and 12.7% reported that during the past month they had felt *‘downhearted or blue’* most or all of the time*.* Almost all clients (97.6%) had at least one lifestyle risk factor and 101 (12.5%) had at least four (Table 3). At baseline 17.2% of participants reported being smokers, 78.5% had insufficient fruit and vegetable dietary intake, 74.0% were overweight or obese, 36.9% had at risk drinking levels. Of those who were able to engage in physical activity, 50.5% had inadequate levels. There were no significant differences between those in the EI and LI groups (Table 3).

### Recall of lifestyle advice and referrals of clients identified with lifestyle risk factors at baseline and 3 months

Only a minority of participants with a SNAPW risk factor recalled having received advice from any source in the 3 months prior to the baseline survey. There was no difference between the EI and LI groups in this measure. However, in the EI sites the overall percentage of clients who reported having received advice from any source at 3 months increased significantly for dietary advice (from 12.9% at baseline to 23.3% at 3 months) and physical activity advice (from 12.5% at baseline to 19.1% at 3 months). There were no significant changes in the LI sites and at 3 months the early intervention group was more likely to report having received diet advice (Table 4).

**Table 4:** Proportion of at risk clients recalling being offered advice or being referred to manage risk factors

	**Baseline**		**3 months**	
	**Early intervention**	**Late intervention**	**Early intervention**	**Late intervention**
	**n /N**	**n/N**	**n/N**	**n/N**
	**%, (95%CI)**	**%, (95%CI)**	**%, ( 95%CI)**	**%, (95%CI)**
**Offered advice from any provider**				
Diet	33/256	31/253	60/257	40 /247
	12.9% (8.6-17.0)	12.3% (8.2-16.3)	23.3 (18.1-28.5)* #	16.2% (11.6-20.8)
Physical activity (for those able to engage in PA)	15 /120	8/91	35/183	20/162
	12.5% (6.6-18.4)	8.8% (3.0-14.6)	19.1% (13.4-24.8)*	12.3% (7.3-17.4)
Smoking	10 /64	16/65	11 /46	11 /47
	15.6% (6.7-24.5)	24.6% (14.1-35.1)	23.9% (11.6-36.2)	23.4% (11.3-35.5)
Alcohol	6 /105	6/93	6 /84	9/75
	5.7% (1.3-10.2)	6.5% (1.5-11.4)	7.1% (1.6-12.7)	12.0% (4.7-19.4)
**Referral for SNAP intervention**				
Diet	31/326	19/298	40 /257	29 /252
	9.5% (6.3-12.7)	6.4% (3.6-9.2)	15.6% (11.0-20.0)*	11.5% (7.6-15.5)
Physical activity (for those able to engage in PA)	7 /120	5/91	21/100	9/78
	5.8% (1.6-10.0)	5.5% (0.8 – 10.2)	21.0% (13.0-30.0)*	11.5% (4.5-18.6%)
Smoking	8 /74	11 /64	9 /62	10 /49
	10.8% (3.7-17.9)	17.2% (7.9-26.4)	14.5% (5.8-23.3)	20.4% (9.1-31.7)
Alcohol	3 /159	4 /138	9 /131	6 /109
	1.2% (0–4.0)	2.9% (0.1-5.7)	6.9% (2.5-11.2) *	5.5% (1.2-9.8)

* Significant change from baseline p < 0.05.

*#* Significant difference between groups p < 0.05.

There were significant increases in reported referrals for diet, physical activity and alcohol from baseline to 3 months in the EI group (from 9.5 to 15.6%, 5.8 to 21.0% and 1.2 to 6.9% respectively). There was no change in the LI group (Table 4). There were no significant differences between groups at three months.

### ‘Readiness to change’ of clients identified with lifestyle risk factors

At baseline, the majority of clients who were at risk were in the contemplation, preparation or action stages of change for weight change, physical activity, improved nutrition or smoking (65%, 67% 59.4%, and 73.% respectively). However, only a minority of those with at-risk alcohol consumption were in the contemplation, preparation or action phases (48.1%). At 6 months there was a significantly greater shift towards higher stages of change in those who were physically inactive, in the EI group compared to the LI group (58.8% vs 27.8%; Chi square = 4.54, *p = 0.032*), Table 5. Readiness to change smoking increased in LI but not the EI group. There were no other significant changes in either group.

**Table 5:** Shift to higher change stage between baseline and 6 months for clients with SNAPW risk factors

**Clients with SNAPW risk factors**	**Increase fruit and vegetable intake**		**Increase physical activity**		**Reduce alcohol consumption**		**Reduce or quit smoking**		**Reduce weight**	
Intervention site	EI	LI	EI	LI	EI	LI	EI	LI	EI	LI
	n = 101	n = 105	n =34	n =18	n = 57	n =42	n = 24	n = 18	n = 96	n = 97
Did not shift to a higher stage of change	66.3%	61.9%	41.2%	72.2%	80.7%	81.0%	70.8%	44.4%	64.6%	55.7%
Shifted to a higher stage of change	33.7%	38.1%	**58.8%**	27.8%	19.3%	19.0%	29.2%	**55.6%**	35.4%	44.3%
Chi square *(one-sided)*	0.439		**4.54**		0.001		2.973		1.60	
	*p = 0.303*		***p = 0.032***		*p = 0.593*		*p = 0. 080*		*p = 0.132*	

Bolded figures represent significant differences between the sites.

### Client self-reported risk factors

Overall, there were no significant differences in risk factors between EI and LI groups at baseline, 3 or 6 months.

However, there were significant increases in diet scores and physical activity between baseline and 3 months and baseline and 6 months in both groups (Table 6). There were no significant changes in smoking, alcohol or self-reported weight.

**Table 6:** SNAP risk factors scores at baseline, 3 and 6 months

	**Number**		**Baseline**		**3 months**		**6 months**	
	**Early**	**Late**	**Early mean 95%CI**	**Late mean 95%CI**	**Early mean 95%CI**	**Late mean 95%CI**	**Early mean 95%CI**	**Late mean 95%CI**
Diet score^a^	195	201	3.98 (3.76-4.20)	3.98 (3.77-4.19)	4.48* (4.20-4.76)	4.30* (4.03-4.57)	4.44* (4.16-4.72)	4.54* (4.23-4.85)
Physical activity score^b^	60	42	1.73 (1.39-2.07)	1.40 (0.99-1.81)	2.32* (1.87-2.77)	2.48* (1.86-3.10)	2.63* (2.15-3.11)	2.74* (2.06-3.42)
Weight (overweight)	77	104	81.0 (78.9-83.1)	80.3 (78.3-82.3)	80.7 (78.2-83.2)	80.4 (78.1-82.7)	81.0 (78.5-83.5)	81.7 (79.4-84.0)
Weight (obese)	112	82	101.4 (97.8-105.1)	102.3 (98.4-106.3)	100.4 (96.6-104.2)	102.3 (96.8-107.8)	100.3 (96.7-103.9)	101.6 (97.1-106.1)

* Significant difference from baseline.

^a^ Total number of serves of fruit and vegetables per day up to maximum of 7.

^b^ scored 0–8, <4 considered at risk.

Multilevel regression analysis showed that being retired had a positive effect on diet (Table 7). Self-reported health had a positive effect on physical activity score. Males, young, unemployed, those with good mental health and poor general health tended to have a negative effect on weight loss. The intervention was not significantly related to diet score, physical activity score and weight at 6 months (Table 7). The multilevel regression model explained 16% and 42% respectively of the total client variance in the diet and physical activity scores respectively.

**Table 7:** Diet and physical activity scores and weight: multilevel regression models

	**Diet score**		**Physical activity score**		**Weight**	
Diet Score (F & V Serves)	beta	SE	beta	SE	beta	SE
Time (1,2,3 = 0, 3 and 6 months)	0.039	0.044	0.003	0.077	-0.107	0.298
Intervention (early)	-0.178	0.125	0.032	0.146	0.142	2.13
Male	-0.776	0.110	0.195	0.133	**5.376**^**†**^	1.605
Age (BL)	0.006	0.007	0.002	0.008	**-0.548**^**†**^	0.090
Employed	0.206	0.170	0.227	0.224	**-13.30**^**†**^	2.300
Retired	0.420*	0.177	-0.157	0.244	2.052	2.460
Wound management	-0.134	0.229	0.279	0.242	-1.013	3.708
Catheter/incontinence	0.055	0.399	0.052	0.468	-6.318	6.884
General post hospital care	0.144	0.315	0.647	0.354	6.700	4.874
Other	-0.334	0.281	0.395	0.321	1.521	4,522
Mental health – good	-0.240	0.133	-0.07	0.169	5.938**	**1.917**
Self-reported good health	0.135	0.118	0.394**	0.146	-5.169**	1.676
No. of health conditions	-0.045	0.031	0.005	0.038	0.185	0.425
No. of risk factors	-0.068	0.049	0.038	0.053	0.377	0.687
Variance explained						
Client level	16.1%		41.6%		0%	
Time level	3.8%		1.3%		15.5%	

* p < 0.05 ** p < 0.01 ^†^ p < 0.001.

SE: standard error.

## Discussion

This study demonstrated that community health nurses were able to implement lifestyle risk factor management as part of normal clinical practice. This individual support within PHC can complement broader population health approaches as part of a comprehensive approach to reducing cardiovascular risk factors across the population. Community health nurses are particularly well placed to deliver lifestyle interventions to high risk clients, many of whom have chronic disease and multiple behavioural risk factors.

The intervention was associated with an increase in the provision of brief diet and physical activity advice by community nurses. In qualitative interviews we found that this was a feasible addition to routine practice by the nurses which clients found acceptable [33,34]. Whilst referrals were infrequent at baseline they increased following the intervention for diet and alcohol and physical activity in the EI but not the LI groups.

Despite modest improvements in preventive care, and some shift in readiness to change physical activity, there was no evidence of a significant impact of the intervention on the SNAP behaviours or weight of clients. It may be that brief interventions from community nurses is not sufficient to achieve change in lifestyle risk factors in this group of clients, many of whom were older, had existing chronic conditions, or were recovering from acute illness. An important factor may also be that many clients were seen following discharge from hospital, and the immediate post-acute phase might not be conducive to making lifestyle change. The intervention and follow-up period in this study was relatively short and it is possible that clients might have been able to make changes once they were fully recovered. These clients may require referral onwards to more intensive interventions at an appropriate time. This requires systems to be in place for assessment of readiness to change and referral to other services. However, this was not captured in the study.

Our negative findings are in contrast with other research in the effectiveness of brief lifestyles interventions in the PHC setting. Most of that research has been conducted in family practice, in services where the nurses were involved in the care of the clients in an ongoing way, or involved major input from referral programs or providers outside PHC [35-38]. However, only a minority of clients of community health nurses in this study received care for longer than 6 months. As has been noted, in the short term many clients had reduced capacity for physical activity because of their illness. Thus while community nurses have the opportunity to assess and initiate behavioural interventions, these need to be provided in the context of long-term care.

Another possible contributor to the negative finding may be related to the relatively high proportion of patients from lower two fifths of socioeconomic disadvantage of many community nursing service clients. This might suggest the need for intervention to address social and environmental factors at the community level. Certainly transport and cost was a major barrier to referral identified by the nurses themselves [33].

Following on from initial assessment and advice, clients who are ready to change need to be linked into longer-term care pathways which support them in changing their risk factors and maintaining them over time. The referral of clients to lifestyle interventions, programs and groups (i.e. Assist, the fourth ‘A’ in the 5As Model) might be a necessary step for many clients to achieve improved health outcomes and reduce risk factors [39,40]. In this study at risk clients infrequently recalled having been referred and other research has identified numerous barriers to referral [41]. The fifth ‘A’, Arrange follow up, is important in the maintenance of behaviour change even over the medium term. Prerequisites for these two actions include adequate availability and affordability of referral services, improved communication, and transfer of care between community health nurses and other providers involved in long-term care. These long-term providers may include the client’s GP, private or public allied health professionals, or other community services and programs. Critical to this transfer is clarity about who is prepared to take on the role of coordinating and monitoring the client’s lifestyle risk factors over months and years.

There are a number of limitations in the study that need to be acknowledged. The data are based on self-report by clients which may have introduced bias especially for weight. Nurses from the LI (comparison) sites commented that simply answering the initial survey prompted them to be more aware of the need to include addressing lifestyle risk factors in their professional care of their clients (i.e. the Hawthorne effect) [42]. This may account for the improvement in physical activity and diet scores in both groups. This study adopted a quasi-experimental design because it was not feasible to randomise the intervention according to individual clients or practitioners within the services. The overall response rate could also have introduced bias affecting the generalisability of the findings, as more interested clients may have chosen to participate.

## Conclusion

The study demonstrated that an intervention to provide community nurse training and support for management of clients’ SNAPW risk factors was associated with increases in advice and referral of clients with risk factors. This was associated with some improvement in client readiness for physical activity. There were no changes, however, in lifestyle behaviours or weight. This suggests a need to facilitate referral to more intensive interventions for clients with risk factors identified by community health nurses and for follow up by providers involved in long-term continuing care. This presents a challenge for the community health care sector and to current practices regarding communication and linkage between primary health care and other services in the Australian health care system.

## Competing interests

The authors declare that they have no competing interest in the conduct of this study.

## Authors’ contributions

All authors contributed to the study design and reviewed and approved the final manuscript.

## Pre-publication history

The pre-publication history for this paper can be accessed here:

http://www.biomedcentral.com/1471-2458/13/375/prepub
